# Cryostructuring of Polymeric Systems: 63. Synthesis of Two Chemically Tanned Gelatin-Based Cryostructurates and Evaluation of Their Potential as Scaffolds for Culturing of Mammalian Cells †

**DOI:** 10.3390/gels8110695

**Published:** 2022-10-28

**Authors:** Vladimir I. Lozinsky, Valentina K. Kulakova, Alexei M. Grigoriev, Elena A. Podorozhko, Ludmila A. Kirsanova, Aleksandra D. Kirillova, Ivan A. Novikov, Yulia B. Basok, Viktor I. Sevastianov

**Affiliations:** 1 A.N.Nesmeyanov Institute of Organoelement Compounds, Russian Academy of Sciences, Vavilov Street 28, Bld. 1, 119334 Moscow, Russia; 2 Institute of Fundamental Medicine and Biology, Kazan (Volga-Region) Federal University, Kremlevskaya Street 18, 420008 Kazan, Russia; 3 V.I.Shumakov Federal Research Center of Transplantology and Artificial Organs of the Ministry of Healthcare of the Russian Federation, Shchukinskaya Street 1, 123182 Moscow, Russia; 4 Scientific Research Institute of Eye Diseases, Rossolimo Street 11A, 119021 Moscow, Russia

**Keywords:** gelatin, cryostructuring, carbodiimide, glyoxal, cell culture scaffolds, in vitro and in vivo bioassay

## Abstract

Various gelatin-containing gel materials are used as scaffolds for animal and human cell culturing within the fields of cell technologies and tissue engineering. Cryostructuring is a promising technique for the preparation of efficient macroporous scaffolds in biomedical applications. In the current study, two new gelatin-based cryostructurates were synthesized, their physicochemical properties and microstructure were evaluated, and their ability to serve as biocompatible scaffolds for mammalian cells culturing was tested. The preparation procedure included the dissolution of Type A gelatin in water, the addition of urea to inhibit self-gelation, the freezing of such a solution, ice sublimation in vacuo, and urea extraction with ethanol from the freeze-dried matter followed by its cross-linking in an ethanol medium with either carbodiimide or glyoxal. It was shown that in the former case, a denser cross-linked polymer phase was formed, while in the latter case, the macropores in the resultant biopolymer material were wider. The subsequent biotesting of these scaffolds demonstrated their biocompatibility for human mesenchymal stromal cells and HepG2 cells during subcutaneous implantation in rats. Albumin secretion and urea synthesis by HepG2 cells confirmed the possibility of using gelatin cryostructurates for liver tissue engineering.

## 1. Introduction

Various gelatin-based and gelatin-containing biopolymeric gel materials are quite popular matrices that are used as scaffolds for animal and human cell culturing within the fields of cell technologies and tissue engineering (e.g., see reviews [1,2,3,4,5,6,7,8]). This trend is attributed to gelatin’s non-toxicity, low immunogenicity, biodegradability, and, as a consequence, excellent biocompatibility [4,5,6,7,8]. For similar implementations of the above-indicated gelatin-based materials, the wide-porous texture of their scaffolds is preferable because it allows for the easy penetration of cells into the matrix bulk and their adhesion to the inner surfaces of pore walls [2,3,4,9]. In connection with this, several approaches for the generation of the required macroporous morphology in these scaffolds have been developed [10,11,12,13]. Among these approaches are the so-called *cryostructuring techniques* [14,15,16,17,18,19,20,21,22,23] that facilitate diverse possibilities for the preparation of wide-pore scaffolds that are well-suited to serve as sponge-like carriers for cell culturing [24,25,26,27,28,29,30,31,32,33,34,35].

In general, the cryostructuring methodology allows for the production of two main types of the resultant polymeric materials [22]. The first ones (***1***) are the so-called *cryogels*—macroporous gel matrices in which the formation of the 3D polymer network occurred in the frozen molecular solutions or colloidal dispersions, respectively, of the precursors [14,18,19,20,23]. With that, the junction knots of the spatial network in various cryogels can be covalent cross-links, low-dissociating ionic/coordination bonds, non-covalent (physical) cross-links such as H-bonds and hydrophobic interactions, or a combinations of the knots of different nature [14,18,19,23,27,36,37,38]. The second type of cryogenically produced materials are the *cryostructurates*—also macroporous polymeric matrices formed as a result of cryogenic processing (the freezing of the initial precursor solutions (variant ***2a***) or gels (variant **2*b***) followed by the removal of the crystallized solvent via sublimation or cryoextraction) when no gelation occurs in a frozen system [16,17,21,23]. Very often, certain post-tanning (cross-linking) of the thus formed matter is required in order to make it insoluble [22,23]. For the preparation of macroporous cryostructurates of the **2*b***-variant, a simple freeze-drying of the preliminary produced gels (usually hydrogels) is employed [22,39]. As a rule, the physico–chemical properties and porosity characteristics of the resultant cryogenically structured polymer materials of the ***2b***-type significantly differ from the same parameters of ***2a***-cryostructurates [22,40,41]. The main reasons for such differences are the rather distinct solvent crystallization conditions in these two cases, since the polymer network of a pre-formed gel interferes with the growth of crystals [42]. Therefore, the properties and macroporous morphology of the cryostructurates produced by the freezing of the liquid and the gel precursor system will differ even if their compositions and the cryogenic processing conditions are formally similar.

A characteristic morphological feature inherent in all types of cryogenically structured polymer matrices is a system of interconnected large pores generated by the polycrystals of the frozen solvent that act as porogens [14,15,16,17,18,19,20,21]. The size and geometry of such pores depend on many factors, mainly the solvent and solutes’ nature, the concentration of the precursors, and the temperature/time regimes of the cryogenic processing [14,17,19,21,23,36,43]. 

The preparation of various gelatin-based cryogels and cryostructurates has been reported [23,27,43,44,45,46,47,48,49], and their promising potential as macroporous, biocompatible scaffolds for cell culturing has been demonstrated [6,32,39,49,50,51,52,53,54,55,56,57]. Nonetheless, there is one significant point related to the features capable of complicating the procedures used for the preparation of the above-mentioned gelatin cryogels and cryostructurates, as well as of similar materials based on other polymers (e.g., agarose [41]), solutions of which undergo the sol–gel transition upon cooling. It is a commonly known phenomenon that aqueous solutions of gelatin quickly turn into a gel-like state at temperatures below about 40 °C [58]. Therefore, during cryogenic treatment, such in situ formed gelatin gels will freeze instead of the respective solutions. In turn, for the effective formation of a wide-pore structure characteristic of cryogels and ***2a***-type cryostructurates, it is necessary to freeze a solution of polymer precursors in order to provide the so-called cryoconcentrating effects to the maximum extent [19]. Since the main mechanism of the sol–gel transformation of aqueous gelatin solutions is the linking of multiple intermolecular hydrogen bonds [58], it is necessary to slow down or, better, suppress such a self-gelation process during the cooling time until solvent crystallization begins. That is why, in the present study, additives of urea were introduced into the initial aqueous gelatin solution with the aim of more or less inhibiting such H bonding. Moreover, we compared two different techniques for the tanning of the ***2a***-type gelatin cryostructurates, namely, the use of either carbodiimide or glyoxal cross-linkers, and then the resultant wide-pore matrices were tested as the 3D carriers for the culturing of mammalian cells. It was also of our interest to compare such gelatin-based sponge-like cryostructurates regarding their physico–chemical characteristics and behavior upon use as cell culture scaffolds.

The potential of gelatin cryostructurates to serve as scaffolds for cell culturing was evaluated with the use of human adipose tissue-derived stem cells (**hADSCs**) and HepG2 liver hepatocellular carcinoma cells. The choice of mesenchymal stem cells (**MSCs**) was defined by their demand in tissue engineering. MSCs have the ability to differentiate in various directions, including chondrogenic, osteogenic, adipogenic, myogenic and neurogenic. In addition, the secretome of MSCs can be used in the treatment of a number of diseases [59,60,61,62,63]. Human hepatoma HepG2 cells were used as the in vitro hepatocyte models due to their availability, ease of handling, high proliferative capacity, and phenotypic stability [64].

By and large, the cryogenically structured gelatin-based scaffolds elaborated in this study were previously unknown, i.e., they have not been reported elsewhere. In this respect, these wide-pore biopolymeric materials are novel, and the operationally simple procedures of their preparation, as well as their physico–chemical properties and suitability for cell technologies, have been developed and explored for the first time. 

## 2. Results and Discussion

### 2.1. Preparation, Properties and Macroporous Morphology of Gelatin-Based Cryostructurates

**Figure 1:**
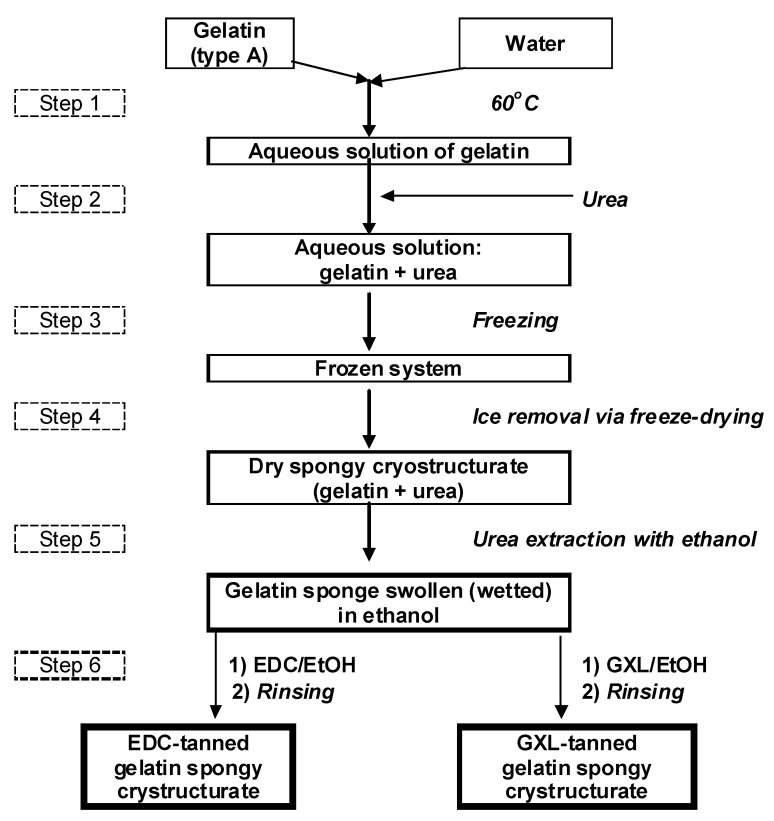
A principal block diagram for the preparation of gelatin cryostructurates.

The EDC- and GXL-tanned gelatin cryostructurates were synthesized according to the block diagram shown in Figure 1 and described in Section 4.2. This rather simple (in terms of manipulations) process consisted of the preparation of the aqueous solution of precursors (gelatin and urea), its freezing, its freeze-drying, the extraction of urea with ethanol (non-solvent for gelatin), the chemical cross-linking (tanning) of the gelatin with EDC or GLX, and the final rinsing of the resultant spongy biopolymeric material with ethanol.

As indicated above (see ‘Introduction’), urea as a H bonding-inhibiting agent was introduced into the feed system (Step 2) in order to suppress the self-gelation of the aqueous gelatin solution during its cooling/freezing. The working concentrations of both solutes, namely, 60 g/L for gelatin and 1 mol/L for urea, were found in the preliminary experiments. On the one hand, these values allowed us to obtain gelatin sponges with sufficient physical integrity and strength for their further use as cell culture scaffolds. On the other hand, such a concentration of chaotropic urea turned out to be sufficient to prevent the self-gelation of the gelatin solution before the onset of freezing. We previously implemented a similar idea for the preparation of cryogenically structured scaffolds on the basis of agarose [41], aqueous solutions of which undergo the sol–gel transition considerably faster than gelatin-containing solutions. In the “agarose case,” the additives of alkali were incorporated into the feed solutions to cause the partial deprotonation of the polysaccharide’s OH groups, thus inducing some repulsion of the equally charged agarose chains. In turn, in the “gelatin case”, a weaker impact of the non-ionic urea turned out to be sufficient to achieve the necessary inhibition of the self-gelation. The conditions of freezing and its duration conditions (−20 °C/18 h) in Step 3 were also optimized in the preliminary experiments.

After the freezing of the precursor solution and the freeze-drying of the frozen samples (Step 4), urea was extracted from the bulk of dry cryostructurates with ethanol (Step 5). All subsequent manipulations with the respective gelatin sponges were carried out in the medium of this solvent, which also enabled sterilization and the maintenance of antimicrobial conditions. The last key stage of the preparation of the scaffolds considered in this study was the solid-phase tanning of these cryostructurates by their treatment with ethanolic solutions of EDC or GXL (Step 6) followed by the final rinsing of the samples with ethanol. As is well known, in the case of EDC, the cross-linking of protein chains occurs via pendant peptide bonds [65], and in the case of GXL, it occurs via aldimine bonds [66].

It was found that the use of the carbodiimide-assisted tanning gave rise to a denser cross-linked 3D network of the polymeric phase (the walls of macropores) within the gelatin cryostructurates compared with the 3D network of the glyoxal-tanned sponges. Upon swelling in water, the former wide-pore materials absorbed a lower total amount of liquid (*S*_tot_) and had a lesser swelling degree of their walls of macropores (*S*_w/w_) than the same parameters for the latter gelatin sponges (Table 1). In addition, the water-swollen EDC-tanned cryostructurates turned out to be more rigid—their apparent compression modulus was higher by a factor of ~2.2 (cf. *E*_app_ values in Table 1).

A quite evident reason for these differences was the higher cross-linking degree of the spatial polymeric network inside the walls of the macropores in the EDC-tanned gelatin sponges in comparison with that of the GXL-tanned ones, since a higher number of such cross-links should lead to a lower swelling capacity of the respective network and, consequently, a higher rigidity of the swollen gel material [67]. Most probably, the observed effect in the different cross-linking efficiency of EDC and GXL is caused by, along with other factors, the significantly different amount of the reactive groups of gelatin macromolecules that could be involved in the formation of interchain cross-links. If one carbodiimide molecule binds one pendant COOH group of aspartate or glutamate residue with one ε-amino group of a lysine monomer unit, glyoxal binds two lysyl residues. That is, only a half of the amount of the interchain links can potentially be formed in the latter case with the participation of the total number of NH_2_ functions in the gelatin chemical structure.

Accordingly, the main indicators of the cross-linking density are the values of the swelling degree of the proper polymer matter, i.e., the values of *S*_w/w_. For the EDC- and GXL-tanned gelatin-based cryostructurates synthesized in this study, the *S*_w/w_ values of, respectively, ~2.5 and ~3.8 g of bound (solvate) water per 1 g of dry polymer (Table 1) mean that the concentrations of gelatin itself in the water-swollen walls of the macropores of the sponges of these two types were approximately 403 and 263 mg/mL. These data testify, respectively, to the 6.7- and 3.5-fold increases in the content of polymer in the phase of hydrated gel walls of the macropores versus the gelatin concentration (60 mg/mL) in its feed solution to be further cryogenically structured. Such an “increase” in the content of polymer matter in the walls of the macropores was the result of the well-known cryoconcentrating effect, when a neat solvent is initially crystallized in the course of non-deep freezing of a feed solution (Step 3, Figure 1) and the solutes are concentrated in the volume of the so-called unfrozen liquid microphase [19,68]. The subsequent freeze-drying of the frozen sample (Step 4, Figure 1) removed the solvent crystals (ice in the case of aqueous systems), thus leaving the macropores in the bulk of the dried matter. In turn, the polymer, which was concentrated in the walls of the macropores, provided the necessary strength of the spongy material and its integrity upon the further swelling in its respective solvating liquid medium.

**Figure 2:**
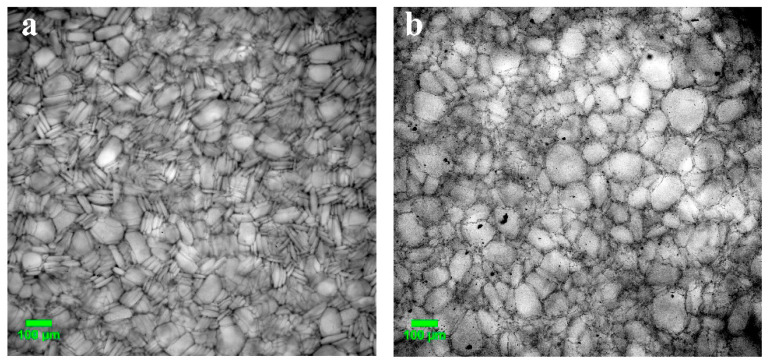
Microphotographs of the water-swollen gelatin-based cryostructurates tanned with EDC (**a**) or GXL (**b**). Optical microscope (see Section 4.4), phase contrast, Giemsa staining; scale bar = 100 μm.

Regarding the macroporous morphology of the gelatin-based cryostructurates prepared according to the processing diagram shown in Figure 1, both the EDC- and GXL-tanned matrices possessed a sponge-like texture with a system of gross pores of 40–220 μm in diameter. The images in Figure 2 are typical examples of the microstructure inherent in such sponges.

The wide pores in a matrix bulk are interconnected, and this morphological peculiarity enables the free penetration of cells into the inner regions of similar cryogenically structured scaffolds upon the stage of cell seeding and the further propagation of growing cells onto the surfaces of the walls of the macropores [12,28,32,69]. It can be seen from the images in Figure 2 that the EDC-cross-linked cryostructurate possessed somewhat smaller pores than the pores in the GXL-tanned sample, and the pore walls in the former gelatin sponge were thinner and clearly defined. Most probably, such morphological differences were determined by the differences in the swelling properties of these polymeric matrices (Table 1). Specifically, the above-discussed lower cross-linking extent of the 3D network of the pore walls in the GXL-tanned cryostructurate was the reason for its higher swelling capacity. As a consequence, in this water-swollen sponge, the walls of the macropores were thicker and the pores themselves were wider.

**Figure 3:**
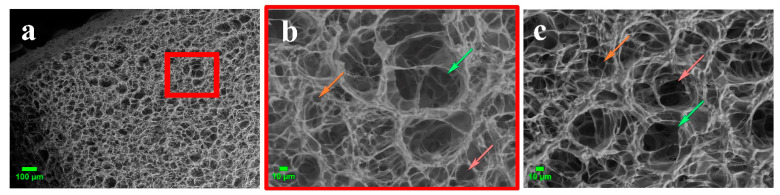
Morphological characterization of the EDC-tanned gelatin cryostructurate with environmental SEM (see Section 4.4): (**a**) the image at low magnification (scale bar = 100 μm); (**b**) the image of the upper surface (scale bar = 10 μm); (**c**) the image of the bottom surface (scale bar = 10 μm). Green arrows—large pores; orange arrows—small pores.

The surface morphology of the gelatin-based cryostructured sponge was studied with the SEM method (Figure 3a–c). In general, the morphology of the sample surface was typical for cryostructurates. Microphotographs show their characteristic feature—macroporosity formed by the polycrystals of a frozen solvent acting as porogens [19]. The surface and subsurface layer of the sample were characterized by an open network of interconnected macropores distributed rather uniformly. The macropores provided the unhindered penetration of cells and transport of oxygen, waste products and nutrients deep into the sponge [11]. A noticeable difference in the macroporous morphology of the upper (Figure 3b) and lower (Figure 3c) parts of the gelatin sponge was observed. During the formation of the cryostructured matrix, the pores in the upper part changed their shape and became larger than the pores in the lower part of the sponge. This was due to the direct contact of the lower surface of the sample with the bottom of the cooled Petri dish, which led to a vertical temperature gradient. Earlier, a very similar trend as registered for the disc-shaped gelatin-based cryostructurates formed in the DMSO medium [48]. Additionally, a similar pattern has been observed for cryostructurates based not only on gelatin but also on other polymers (agarose, serum albumin, etc.) [40,41,70]. These facts evidently testify to the general character of the effects.

From these SEM microphotographs, we determined (by using Image J software) the pore size on the upper and bottom sides of the gelatin discs. Then, depending on their dimension, the pores were classified for each side of a sponge as large (64.1 ± 15.6 µm and 58.3 ± 11.4 µm), medium-sized (29.2 ± 7.7 µm and 23.6 ± 5.9 µm) and small pores (9.5 ± 3.0 µm and 9.9 ± 2.2 µm). These values were somewhat less than those of the cross-section of macropores observed with an optical microscope for the same water-swollen sponges (Figure 3). Most probably, such differences were due to certain contraction of the swollen sponges under the conditions of a “weak” vacuum during the SEM experiments (see Section 4.4). Nonetheless, as was pointed out above, the large pores (in our case, detected with various microscopy methods) were able to ensure the migration of cells into the bulk of the sponge, while the medium- and small-sized pores supported the effective mass transfer of nutrients and gases.

### 2.2. Evaluation of the Gelatin-Based Cryostructurates as Scaffolds for Culturing of Mammalian Cells 

#### 2.2.1. Cytocompatibility of the EDC- and GXL-Tanned Gelatin-Based Cryostructurates

It is commonly recognized that well-designed scaffolds provide favorable environment for the proliferation and differentiation of MSCs and induce some mechanical stimulation, which is attractive for the further use of MSCs and can positively affect the outcome of MSCs therapy [71]. With that, the cytocompatibility of any scaffold is of key significance. Therefore, we explored this aspect for both the EDC- and GXL-tanned gelatin sponges synthesized in the present study.

**Figure 4:**
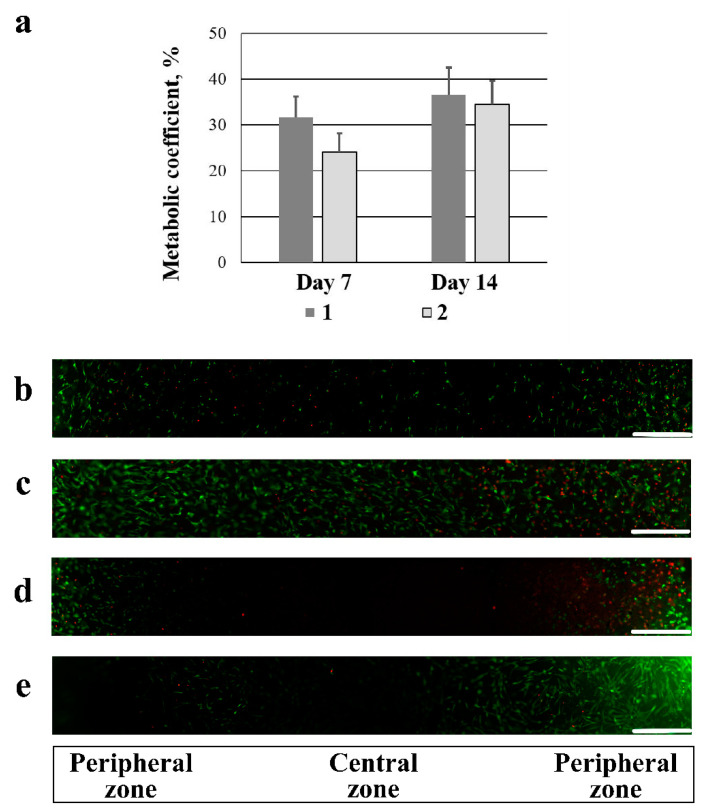
Viability and proliferation of hADSCs adhered onto the EDC- and GXL-tanned gelatin scaffolds: (**a**) hADSC proliferation on the EDC- and GXL-tanned gelatin-based cryostructurates (**1**—EDC-tanned scaffold; **2**—GXL-tanned scaffold); (**b**) hADSC viability on day 7 on the EDC- tanned scaffold; (**c**) hADSC viability on day 7 on the GXL-tanned scaffold; (**d**) hADSC viability on day 14 on the EDC- tanned scaffold; (**e**) hADSC viability on day 14 on the GXL-tanned scaffold. Live cells were green-stained using calcein-AM, and dead cells were red-stained using ethidium homodimer. Scale bar = 500 µm.

It was found that all the samples supported the adhesion and proliferation of hADSCs (Figure 4a–e). The results of the analysis of the metabolic activity of the cells (Figure 4a) demonstrated that there was no significant difference in cell proliferation on both carriers on the 7th and 15th days of culturing. Fluorescence microscopy showed that the live green cells were initially more actively attached in the peripheral zone of the carrier and, further, were spread over the central zone, evenly populating the entire area of the scaffold by 14 days of culturing. By the 7th and 14th days of culturing, only single red-colored dead cells were observed in both samples (Figure 4b–e). The presence of an insignificant number of dead cells is not a scaffold disadvantage, since it is similar to the physiological processes occurring during tissue homeostasis [72].

Thus, the study demonstrated that EDC- and GXL-tanned gelatin-based cryostructurates did not exhibit cytotoxic properties and showed cytocompatibility—they supported the adhesion and proliferation of hADSCs.

#### 2.2.2. Proliferation and Liver Function Studies of HepG2 Cells upon Culturing in Gelatin-Based Cryostructurates

**Figure 5:**
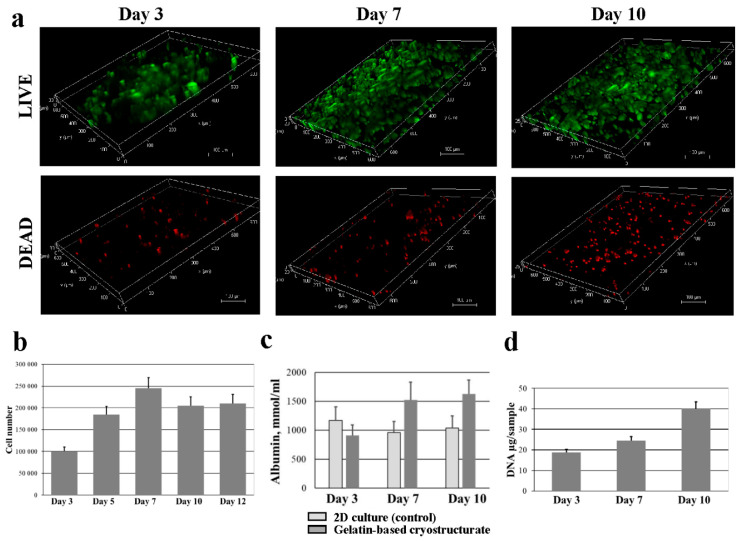
Proliferation and liver function of HepG2 cells attached to gelatin-based cryostructurate. (**a**) HepG2 viability at 3, 7, 10 days on gelatin gelatin-based cryostructurate. Live cells were green-stained using calcein-AM, and dead cells were red-stained using ethidium homodimer. Scale bar 100 µm; (**b**) HepG2 proliferation on gelatin gelatin-based cryostructurate (scale bar 100 µm); (**c**) Albumin secretion; (**d**) DNA content.

The development of 3D in vitro models for the detection of the chronic hepatotoxicity of drugs has shown greater effectiveness than 2D cultures [73,74]. To establish the ability of our gelatin-based cryostructurates to be the basis for creating a 3D model for pharmacological studies, we used HepG2 cells since they perform liver cell-specific functions [75,76]. To study the viability of HepG2s on gelatin-based cryostructurates, living and dead cells were detected using fluorescence microscopy (Figure 5a).

On day 1, the proportion of the green-colored living cells surpassed the number of dead cells. The same pattern was observed at all periods of observation, and the number of both living and dead cells increased with time. The analysis of the metabolic growth curve showed that a significant number of cells were attached to the gelatin-based cryostructurate (Figure 5b). From 3 to 7 days, a phase of logarithmic growth was observed—the number of cells in the scaffold increased by a factor of 2.5 in 4 days, up to 250,000 cells per sample. From the 7th to 12th days of culturing, the cell growth was inhibited, and in general, a plateau was observed—the number of actively metabolizing cells slightly decreased due to the natural aging of the cell culture and, possibly, the exhaustion of the populated space in the gelatin sponge. However, the number of cells on days 7, 10 and 12 did not significantly differ.

Albumin secretion and urea synthesis are two important markers for assessing a specific liver function. The level of the albumin secretion of HepG2 cells cultured for 3 days, 7 days and 10 days in a 2D culture and in the spongy carrier is shown in Figure 5c. Albumin secretion was assayed by ELISA. The obtained data indicate the ability of HepG2s seeded on a gelatin-based cryostructurate to maintain the cells’ secretory function, starting from 6 days of culturing, at a higher level than in the 2D culture. The production of albumin by cells in the cryogenically structured scaffold sharply increased on the 6th day of culturing. At the same time, no significant difference in the albumin content on the 3rd day of culturing between in the control and experiment was observed. The functional activity of the studied cell-scaffold construct for the production of urea from ammonia increased with the time of culturing. On day 3, the urea content in the culture medium was lower than the assay range; on day 7, it was 1.2 ± 0.2 mmol/L; and by day 10, it increased to 1.8 ± 0.4 mmol/L. At the same time, the level of urea concentration during cell culture in suspension remained relatively constant (1.1–1.2 mmol/L) for 10 days. 

The increase in the rate of ammonia metabolism can be explained by the proliferation of cells upon their culturing in the used wide-pore gelatin scaffold. In addition, the amount of DNA isolated from the samples with the HepG2 cells increased during the culturing period (Figure 5d), thus directly showing an increase in the number of cells in the scaffold. In other words, the HepG2 cells upon culturing in the gelatin scaffold proliferated and exhibited certain specific functions of hepatocytes.

In general, the used scaffold, i.e., the EDC-tanned gelatin-based cryostructurates in this case, supports the 3D culture of the HepG2 cells, thus indicating the potential of their use in drug screening and liver tissue engineering [77,78,79].

### 2.3. In Vivo Biocompatibility of Gelatin-Based Cryostructurates

In order to evaluate the biocompatibility of the developed gelatin-based sponges, the samples of the EDC-tanned cryostructurates were implanted into the thigh of laboratory rats (see Section 4.9). After 21 days, the subcutaneously implanted sponges were extracted together with surrounding tissues for a histological examination. During the entire observation period, there were no signs of the development of the inflammatory process in rats of both the control and experimental groups, and wound healing took place by primary intention.

**Figure 6:**
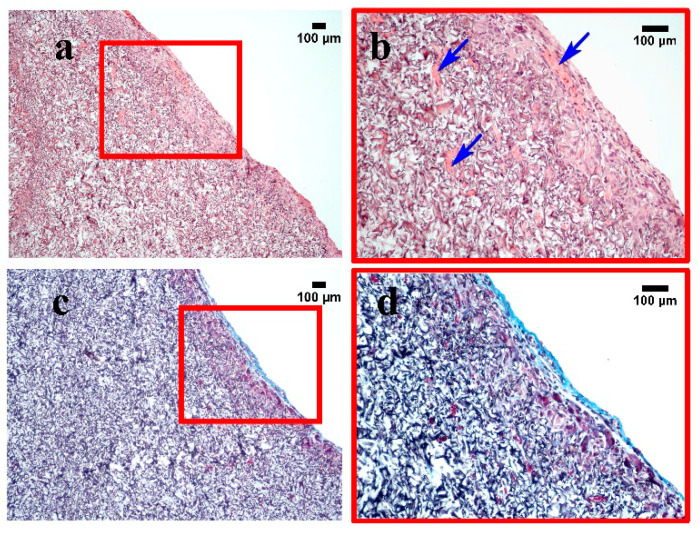
Representative images of the gelatin sponge on the 21 day of subcutaneous implantation: (**a**,**b**) hematoxylin and eosin staining; (**c**,**d**) Masson staining. Scale bar = 100 µm. Blue arrows—blood capillaries.

A thin-walled capsule of connective tissue was highlighted along the border of the sample (Figure 6). A significant number of foreign body giant cells was present. The growth of blood capillaries was also observed in a scaffold (Figure 6b). 

Masson staining showed the presence of collagen only in the area of the connective tissue capsule (with a thickness of less than 100 µm) (Figure 6c,d). Such a result is known to be typical for the in vivo response to the implantation of a “biocompatible” biomaterial because of a mild inflammatory reaction that, after 3–6 weeks, resolves itself into a thin fibrous capsule (in fact, a scar) [80,81]. In general, the results obtained in the in vivo testing of the gelatin-based cryostructurates confirmed their biocompatibility.

## 3. Conclusions

At present, diverse biocompatible, non-toxic, and biodegradable gelatin-based scaffolds are considered to be promising materials for the purposes of cell technologies and tissue engineering. In many cases, similar scaffolds should possess a wide-pore texture, and the pores themselves should be interconnected. In this regard, the cryostructuring of polymeric systems is an attractive and prospective technique. In the present study, two examples of novel gelatin-based materials, namely, spongy cryostructurates, were prepared and characterized, and their potential to perform as efficient scaffolds for the culturing of mammalian cells was evaluated. These scaffolds were synthesized by freezing an aqueous solution containing a mixture of Type A gelatin and urea (the inhibitor of H bonding and the self-gelation of gelatin solutions), freeze-drying the samples, and performing urea extraction with ethanol from the freeze-dried matter followed by its tanning in an ethanol medium either with carbodiimide or with glyoxal. Optical microscopy and SEM data testified to the interconnected wide-pore morphology of such cryogenically structured gelatin sponges. It was shown that the use of carbodiimide gave rise to a denser cross-linked polymeric 3D network in the walls of the macropores of the resultant spongy scaffold versus the use of glyoxal-induced tanning. In the former case, the swelling degree of the polymer network was about 1.5 times lower than that in the latter case, and, as a consequence, the apparent compression modulus of the water-swollen EDC-tanned cryostructurate was higher by a factor of ~2.2 than the modulus of the swollen GXL-tanned sponge. The in vitro and in vivo evaluation of the biocompatibility of the synthesized cryostructurates, their cytotoxicity, and suitability to operate as effective wide-pore scaffolds for the 3D culturing of mammalian cells showed that these materials may be promising scaffolds in tissue engineering. Human mesenchymal stromal cells and HepG2 cells proliferation in vitro and the typical in vivo response to the subcutaneous implantation of a “biocompatible” biomaterial indicate that gelatin cryostructurates may be applicable in future clinical use. When cultured in gelatin cryostructurates, the HepG2 cells secreted an amount of albumin 1.6 times greater and increased the level of ammonia metabolism by 1.5 times versus the culturing in a monolayer culture. Thusly, the synthesized cryostructurates appear to be a useful tool for in vitro toxicological and pharmacological studies on HepG2 cells. In the near future, such scaffolds will be biotested using other cell types—HepaRG, primary human hepatocytes, or pig hepatocytes in particular, all in order to confirm the possibility of the use of similar scaffolds for tasks in, for instance, pharmacology, particularly in drug screening.

## 4. Materials and Methods

### 4.1. Chemicals

The following substances and reagents were used in the experiments without additional purification: 300 Bloom gelatin of Type A derived from porcine skin collagen, urea (ultra-pure grade), N-(3-dimethylaminopropyl)-N′-ethylcarbodiimide) (**EDC**), and ammonium chloride—all from Sigma-Aldrich Inc. (St. Louis, MO, USA); a 40% aqueous solution of glyoxal (**GXL**) and a 96% aqueous solution (*v*/*v*) of ethanol—both from Reakhim (Moscow, Russian Federation). Dulbecco’s modified Eagle’s medium (Nutrient Mixture F-12 (DMEM/F12), alanyl–glutamine and Giemsa dye were purchased from PanEco (Moscow, Russian Federation). Fetal bovine serum (FBS), antibiotic/antimycotic, and HEPES were purchased from Gibco Inc. (Billings, MT, USA). Basic fibroblast growth factor (bFGF) was purchased from Peprotech (Cranbury, NJ, USA). A Human Albumin ELISA Kit, Picogreen QuantiT™, Live/Dead^®^ Cell Viability/Cytotoxicity Kit and PrestoBlue™ Cell Viability Reagent were purchased from Invitrogen Corp. (Carlsbad, CA, USA). DNeasy Blood & Tissue Kit was obtained from QIAGEN (Hilden, Germany). A neodymium-containing contrasting solution for scanning electron microscopy (SEM) was obtained from Glaucon LLC (Moscow, Russian Federation). All aqueous solutions were prepared using deionized water of Milli-Q quality generated with a Simplicity Water Purification System (Millipore, Molsheim, France).

### 4.2. Preparation of the Cryogenically Structured Gelatin Scaffolds

A known amount of dry gelatin powder was dispersed in a calculated volume of water to reach a polymer concentration of 60 mg/mL. Then, the mixture was stirred at 60 °C until the completion of gelatin dissolution. Subsequently, the required amount of dry urea was added and dissolved in this liquid system in such a way that the urea concentration in the resultant solution was 1 mol/L. Next, the warm (~40 °C) solution was poured as the 2 mm in height layer in plastic Petri dishes (inner diameter of 38 mm; OAO Medpolimer, Moscow, Russian Federation) or in 24-well cell culture plates (Corning Inc., Kennebunk, ME, USA) that were quickly placed on a strictly horizontal cooling metal plate equipped with a F-32 ME (Julabo, Seelbach, Germany) programmable cryostat camera, where the samples were frozen and incubated at −20 °C for 18 h. Thereafter, the samples were freeze-dried for 18 h in a vacuum (0.04 mBar) using an Alpha 1-2 LD plus freeze-drier (Martin Christ, Osterode am Harz, Germany). The urea from the resultant dry discs was then extracted with ethanol at room temperature until the absence of the solutes in the liquid phase, as checked with an evaporation test. Then, the rinsed spongy gelatin cryostructurates were treated at room temperature for 48 h with 0.05 mol/L ethanolic solutions of either EDC or GXL and subsequently rinsed with pure ethanol until the absence of the solutes in the liquid phase. Final discs before their use were stored at 4–6 °C under a layer of ethanol.

### 4.3. Physico–Chemical Properties of the EDC- and GXL-Tanned Gelatin Cryostructurates

The swelling parameters of the gelatin sponges were evaluated using the gravimetric technique. To this end, the ethanol from the polymeric discs was removed with multiple rinses in water. Then, the swollen samples were taken out of water, free moisture was carefully removed from the surfaces of the sponges by blotting with filter paper, and the samples were weighed, thus determining the total mass (*m*_swn_) of the swollen material. After that, each swollen sample was placed on a glass Schott filter, and a plastic Petri dish containing a 100 g kettlebell was installed on the top of a water-swollen matrix; under this load, the capillary liquid was removed from the sponge in a vacuum (~15 mmHg) for 5 min. The resultant pressed-off wet disk was weighed, thus giving the value of the swollen polymer’s mass (*m*_wet_), and after subsequent drying (an SNOL 24/200 air thermostat, AB Utenos Elektrotechnika, Vilnius, Lithuania) at 105 °C to a constant weight, the mass of the dry polymer (*m*_dry_) was found. Using the measured values, the total swelling capacity (*S*_tot_) of the respective cryostructurates and the swelling degree (*S*_w/w_) of their polymeric core were calculated as follows:

*S*_tot_ = *m*_swn_:*m*_dry_ (g of H_2_O (bound + capillary)/g of dry polymer) (1)

*S*_w/w_ = (*m*_wet_ − *m*_dry_):*m*_dry_ (g of bound H_2_O/g of dry polymer) (2)

**Figure 7:**
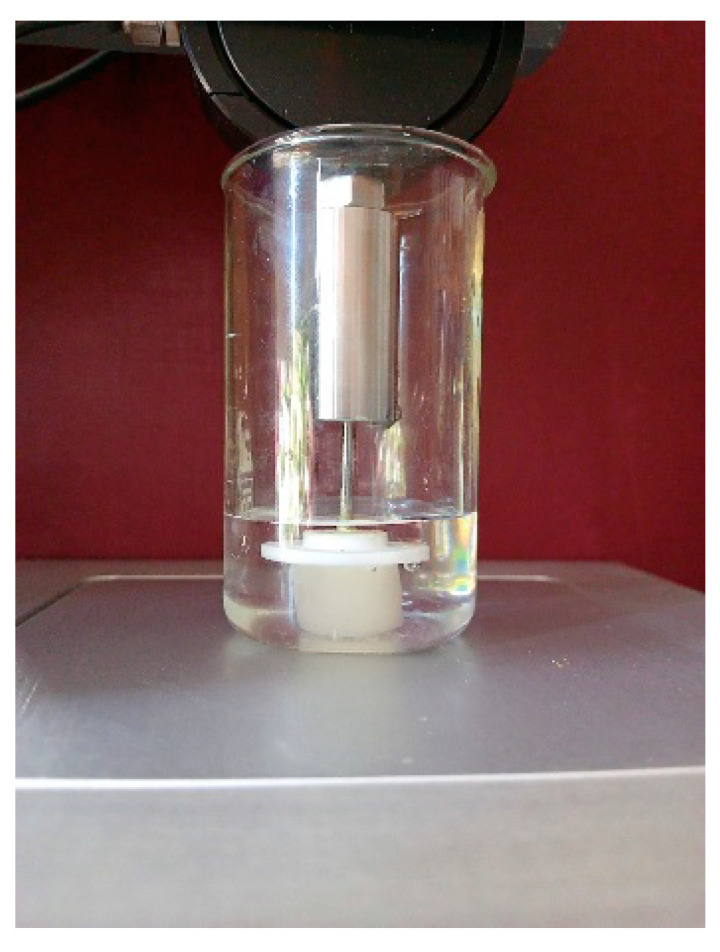
Spongy gelatin cryostructurate uniaxially compressed with a plate-like punch connected to a TA-Plus texture analyzer.

The measurements of the elastic characteristics of the water-swollen gelatin cryostructurates were performed using the cylindrical spongy samples that were 16 mm in diameter and 20 mm in height. Such measurements were accomplished in accordance with a technique that was previously employed for a case of soft spongy cryogels produced from the aqueous solutions of locust bean gum [82]. During our experiments, each swollen sample was placed in a glass beaker filled with water in order to compensate the loss of capillary pressure upon water squeezing from the capillaries following the uniaxial compression. A ‘plate-like’-type punch with a diameter of 10 mm and a thickness of 2 mm was used to minimize the action of the buoyancy of the liquid on the punch connected to the holder of an a TA-Plus automatic texture analyzer (Lloyd Instruments, West Sussex, UK) (Figure 7). Compression was performed at a loading rate of 0.3 mm/min until 50% deformation, and the value of the compression modulus was determined by the instrument-built software. Since the rigidity of similar swollen heterophase spongy materials is the sum of the strength of the polymeric phase (swollen gel walls of macropores) and capillary forces, the physical sense of the measured elastic modulus values somewhat differs from that of homophase gels. Therefore, below, with regard to the gelatin cryostructurates, we call these values the apparent instantaneous compression moduli—*E*_app_.

The *S*_w/w_ and *E*_app_ values were measured for 3–5 parallel samples in three independent experiments. The obtained results were averaged. 

### 4.4. Microstructure of Gelatin-Based Cryostructurates

The microstructural studies of the gelatin cryostructurates in the water-swollen state were performed using optical microscopy in a phase contrast mode and environmental SEM. In the former case, the gelatin-based cryogenically structured samples were the macroporous discs with a diameter of 6 mm and a thickness of 2 mm, which were cut using a punch for a biopsy (Sterylab, Milan, Italy), and a Leica 8DMi Thunder optical microscope (Leica microsystems, Wetzlar, Germany) was used for the microstructural analysis. The spongy samples were stained for 30 min with a working solution of Giemsa stain prepared from a commercially available stock solution according to recommendations of the manufacturer. The samples were then washed under a stream of water. In the SEM case, the morphology of the surface and the nearest subsurface layers of the samples was studied by applying preliminary lanthanide contrasting (see Section 4.1) in accordance with the manufacturer’s instructions. This treatment protocol included the primary flushing of a sample, its incubation for 45 min in a BioREE-A contrasting solution, its rinsing with distilled water, and the removal of excess moisture from its surface. Then, the sample was placed on the microscope table of an EVO LS10 environmental scanning electron microscope (Carl Zeiss NTS, Oberkochen, Germany). The observations were conducted with a backscattered electron (BSE) detector in a low vacuum mode (EP, 70 Pa) at an accelerating voltage of 20–25 kV. The average pore size of the gelatin cryostructurates was determined using Image J software (National Institutes of Health, Bethesda, MD, USA) by measuring 90 randomly selected pores on SEM images.

### 4.5. In Vitro Cell Culture

Adipose tissue samples weighing 3–5 g were obtained with the informed consent of a living healthy donor during liver transplantation under general anesthesia (*n* = 1). The manipulations were performed in accordance with the guidelines of the Helsinki Declaration and approved by the local Ethics Committee at the Shumakov National Medical Research Center of Transplantology and Artificial Organs, Moscow, Russian Federation (15 November 2019, Protocol No.151119-1/1e). The hADSCs, isolated according to the previously developed method and characterized before use, were stored in liquid nitrogen at −196 °C [83]. After defrosting, hADSCs and HepG2 cells taken from the collection of Shumakov cell cultures of the National Medical Research Center for Transplantology and Artificial Organs were cultured at 37 °C in a 5% CO_2_ in a growth medium (DMEM/F12 medium with the addition of 10% FBS, 10 µg/mL of bFGF, antibiotic and antimycotic, 1 mM of HEPES, and 2 mM of alanyl–glutamine). The growth medium was changed every 3 days.

### 4.6. Live/Dead Assay

A total of 5.0 **×** 10^5^ HepG2 cells or hADSCs were seeded onto each scaffold disc of 2 mm in thickness and 6 mm in diameter. Cell viability was assessed with the standard protocol of a live/dead assay. Briefly, the samples were soaked in a staining solution at 37 °C for 30 min. The solution consisted of 1 µM of calcein AM and 0.1 mM of propidium iodide prepared in PBS. The samples were washed twice with PBS and then studied under a Leica 8DMi Thunder optical microscope supplied with LAS X software (Leica microsystems, Wetzlar, Germany) using λ_ex_ = 490/λ_em_ = 515 nm for calcein AM and λ_ex_ = 495/λ_em_ = 635 nm for ethidium homodimer.

### 4.7. Prestoblue Assay

First, 1.0 **×** 10^5^ HepG2 cells or 5.0 × 10^5^ hADSCs were seeded onto each scaffold disc of 2 mm in thickness and 6 mm in diameter. PrestoBlue™ Cell Viability Reagent was used for the cell viability assay, which was carried out in a 96-well plate according to the manufacturer’s instructions. Briefly, the measurement of cell viability was based on the conversion of resazurin into a resorufin product detectable by a spectrophotometer. After the incubation of the cells with 100 μL of the PrestoBlue reagent at 37 °C/5% CO_2_ for 3 h, the light absorbance was measured at 570/600 nm using a Spark 10M microplate reader (Tecan Trading AG, Männedorf, Switzerland). The assay was performed on HepG2 cells cultured on a scaffold for various time periods (days 1 to 10). The absorption measurement data were used to calculate the metabolic activity coefficient (*K*) according to the equation [83]:

(3) $$K=\frac{117.216\;\times \;Abs_{570}\;-\;80.586\;\times \;Abs_{600}}{155.677\;\times \;Abs_{600}\;-\;14.652\;\times \;Abs_{570}}\%$$

where *Abs*_570_ is the absorption at 570 nm and *Abs*_600_ is the absorption at 600 nm. The number of cells was determined according to a calibration curve, which was based on the values of metabolic activity coefficients corresponding to known cell numbers.

### 4.8. Liver Specific Functions Assays

A total of 5.0 **×** 10^5^ HepG2 cells was seeded onto each scaffold disc of 2 mm in thickness and 6 mm in diameter. The resulting bioconstructs were cultured in a growth medium under standard conditions for 15 days. On days 3, 7, and 10, the albumin content in the culture medium was examined by the ELISA assay. The rate of ammonia metabolism was evaluated after 90 min of incubation with 1 mM of ammonium chloride in the culture medium on the 3rd, 7th, and 10th days of the experiment. The amount of urea in the medium was measured using a KonelabPrime 60i biochemical analyzer (ThermoFisher Scientific, Vantaa, Finland). As a control, the culture medium from the cells that were cultured in the same amount in a 6-well cell culture plate (Corning Inc., Kennebunk, ME, USA) was used. The cell proliferation in the scaffolds was assessed by the amount of DNA in the samples. The DNA was isolated from the samples using the DNeasy Blood & Tissue Kit (QIAGEN, Hilden, Germany) according to the manufacturer’s instructions. The DNA content was then measured using the Quant-IT PicoGreen dsDNA Assay Kit (Invitrogen Corp, Carlsbad, CA, USA) in accordance with the manufacturer’s specifications using a Spark 10M microplate reader (Tecan Trading AG, Männedorf, Switzerland). Seven samples were collected at each time point.

### 4.9. In Vivo Studies

Six-month-old Wistar rats (males) weighing 490–550 g (*n* = 9) were used in the experiments. The manipulations did not cause pain to the animals and were carried out in compliance with the following Russian legislation: GOST 33215-2014 (Guidelines for accommodation and care of laboratory animals. Rules for equipment of premises and organization of procedures) and GOST 33216-2014 (Guidelines for accommodation and care of laboratory animals. Rules for the accommodation and care of laboratory rodents and rabbits). The work was approved by the local Ethics Committee at the Shumakov National Medical Research Center of Transplantology and Artificial Organs, Moscow, Russia (5 February 2021, Protocol No. 050221-1/2e). The duration of quarantine (acclimatization period) for all animals was 14 days.

All animals were randomly divided into 2 groups: 1 group (control) of rats that were opened but without implantation (sham operation) (*n* = 3) and 1 group of rats with the implantation of EDC-tanned gelatin cryostructurates (*n* = 6). After being cut into 25 mm^2^ fragments, scaffolds were washed in Milli-Q water for 1 day at room temperature. The materials were subcutaneously implanted in the thigh of each rat. Anesthesia was induced by the intramuscular administration of 15 mg/kg of Zoletil 100 (Virbac, Carros, France). The openings were then sutured with resorbable sutures Vicryl 6.0 (Ethicon, Somerville, NJ, USA). After surgery, no antibiotic treatment was administrated. The duration of animal follow-up after the sample administration was 3 weeks.

### 4.10. Statistical Analysis

Data were analyzed with SPSS26.0 statistical software package. The results are presented as a mean ± SD. The distribution of the variables was tested with the Shapiro–Wilk procedure. The results were compared using *t*-test, one-way analysis of variance (ANOVA), and the Tukey’s honestly significant difference post hoc-test. When *p* < 0.05, a result was considered as a statistically significant.

## Figures and Tables

**Table 1:** Swelling parameters and apparent compression moduli of the EDC- and GXL-tanned gelatin-based cryostructurates.

	Physico–Chemical Characteristics of Gelatin Sponges		
Tanning Reagent	*S*_tot_ (g of H_2_O (Bound + Capillary)/g of Dry Polymer)	*S*_w/w_ (g of Bound H_2_O/g of Dry Polymer)	*E*_app_ (kPa)
EDC	41.1 ± 3.3	2.48 ± 0.26	11.6 ± 0.5
GXL	48.6 ± 4.6	3.80 ± 0.28	5.18 ± 0.35

## Data Availability

The data presented in this study are available on request from the corresponding author.
